# The evolution of three-dimensional knee kinematics after ACL reconstruction within one year

**DOI:** 10.3389/fbioe.2025.1572160

**Published:** 2025-04-23

**Authors:** Lingchuang Kong, Huahao Lai, Xiaolong Zeng, Peng Gao, Wenhao Liang, Qi Gao, Zhiyuan Kong, Wu Wu, Xiaona Wu, Tao Zhang

**Affiliations:** ^1^ Department of Orthopedics, General Hospital of Southern Theatre Command of People’s Liberation Army, Guangzhou, China; ^2^ Department of Bone and Joint Rehabilitation, Guangdong Work Injury Rehabilitation Hospital, Guangzhou, Guangdong, China; ^3^ Department of Orthopedics, Guangdong Provincial Hospital of Chinese Medicine, Guangzhou, China; ^4^ Department of Graduate School, Guangzhou University of Chinese Medicine, Guangzhou, China; ^5^ Department of Neurosurgery, General Hospital of Southern Theatre Command of People’s Liberation Army, Guangzhou, China

**Keywords:** anterior cruciate ligament reconstruction, knee kinematics, gait analysis, postoperative rehabilitation, knee

## Abstract

**Introduction:**

This study aims to explore the dynamic changes in the six degrees of freedom (6DOF) kinematics of the knee joint in patients within one year after anterior cruciate ligament reconstruction (ACLR), combined with clinical scoring systems to analyze functional recovery characteristics, providing scientific evidence for optimizing postoperative rehabilitation strategies.

**Methods:**

The study enrolled 49 patients followed up at 3 months postoperatively, 33 patients at 6 months, and 35 patients at 12 months. Twenty-nine healthy controls were recruited. A three-dimensional motion capture system was used to collect 6DOF knee kinematic data at 3, 6, and 12 months after surgery, including flexion-extension, internal-external rotation, adduction-abduction angles, and anterior-posterior, distal-proximal, medial-lateral translation data. Clinical function was assessed using the IKDC and KOOS scores. One Way ANOVA of one-dimensional statistical parametric mapping (SPM1D) was used to assess the changes in gait kinematics and differences compared to healthy controls.

**Results:**

After ACLR, the IKDC and KOOS scores of patients significantly improved between 3 and 12 months postoperatively, showing good subjective functional recovery. Over the course of one year, the knee kinematic data of gait has gradually recovered. However, abnormalities in knee joint kinematics still exist. In the coronal plane, the adduction angle of the knee joint during motion is relatively large (p < 0.05); In the sagittal plane, the flexion angle increased during the standing phase (p < 0.05); In the transverse plane, the internal rotation angle of the knee joint increased compared to the controls (p < 0.05). The range of motion of flexion and rotational angles decreased compared to the controls (p < 0.05).

**Discussion:**

The kinematic recovery of the knee joint in ACLR patients presents multidimensional characteristics and dynamic changes. The recovery rates and patterns differ significantly across dimensions, with some abnormalities not fully corrected within one-year post-urgery. These findings provide scientific evidence for individualized rehabilitation strategies, emphasizing the need for strengthening joint stability and range of motion recovery in the early postoperative phase (0-6 months) and focusing on correcting rotational and flexion-extension function during the later phase (6-12 months) to further improve knee function and prevent long-term adverse outcomes.

## 1 Introduction

Anterior cruciate ligament reconstruction (ACLR) is a common surgical procedure to restore knee stability and function following ACL injury (Lohmander et al., 2007; Rodriguez-Merchan and Encinas-Ullan, 2022). Despite advancements in surgical techniques, achieving optimal postoperative recovery remains challenging, as it involves complex rehabilitation to restore not only static stability but also dynamic functional movements. A critical aspect of ACLR rehabilitation is understanding the recovery trajectory of knee joint kinematics, particularly in terms of the six degrees of freedom (6DOF), including flexion-extension, internal-external rotation, adduction-abduction, and translation across the anterior-posterior, medial-lateral, and distal-proximal axes (Zhang et al., 2015). These kinematic changes are essential for assessing the progression of functional recovery and the restoration of normal joint motion.

Previous studies have predominantly focused on clinical outcomes and static measurements (Elabd et al., 2024; Hamido et al., 2021; Herbst, et al., 2023), often neglecting the dynamic kinematic behavior of the knee during functional movements, such as walking or running. However, dynamic kinematic analysis provides crucial insights into the joint’s functional performance, which is not fully captured by traditional clinical scoring systems like the International Knee Documentation Committee (IKDC) or the Knee injury and Osteoarthritis Outcome Score (KOOS) (Holsgaard-Larsen et al., 2014; Kaiser et al., 2017; Lepley and Kuenze, 2018). Static measurements, such as clinical scores or radiographic evaluations, primarily assess joint stability at rest but fail to capture transient kinematic changes during functional movements like gait. In contrast, dynamic analysis enables the identification of phase-specific abnormalities that correlate with functional deficits, offering insights into compensatory mechanisms and targeted rehabilitation needs (Yang et al., 2019; Zhang et al., 2016), underscoring the need for kinematic monitoring. Understanding how kinematic parameters evolve over time after surgery is vital for optimizing rehabilitation strategies that are both individualized and comprehensive.

This study aims to fill this gap by investigating the dynamic changes in knee joint kinematics across 1 year post-ACLR surgery, using a three-dimensional motion capture system to evaluate 6DOF knee motion. The study also integrates clinical scoring systems (IKDC and KOOS) to assess functional recovery, thus offering a multidimensional view of post-surgical knee rehabilitation. By analyzing the kinematic patterns at multiple time points (3, 6, and 12 months) and comparing them to healthy controls, this research seeks to provide valuable insights into the recovery process and inform more targeted, evidence-based rehabilitation strategies for ACLR patients.

## 2 Materials and methods

### 2.1 Subjects recruitment

This study recruited 49 ACLR patients followed up at 3 months postoperatively, 33 ACLR patients at 6 months, and 35 ACLR patients at 12 months. All the subjects underwent single-bundle anatomical ACLR by the orthopedic team of the hospital under the same technical standards and received standard rehabilitation treatment within 1-year post-surgery. Subjects were required to be aged between 18 and 50 years, and able to complete all research-related tests and follow-up visits. Exclusion criteria included severe postoperative complications such as infection, fractures, or the need for reoperation, the presence of other knee joint diseases such as meniscal tears or knee osteoarthritis, any other lower limb-related injuries within 1-year post-surgery, undergoing other interventions that may affect knee function, and neurological diseases or other major health issues that could interfere with the study.

This study enrolled 29 control subjects. Participants in the control group were required to be aged between 18 and 50 years, with no history of lower limb-related injuries or surgeries, no history of chronic pain or long-term medication use, and the ability to complete all research-related tests and follow-up visits. Exclusion criteria included any acute or chronic diseases affecting muscle function, undergoing medication treatments that could influence muscle size or function, any lower limb-related injuries in the past 6 months, and neurological disorders or conditions affecting joint movement. All the subjects were consented to participate by signing the informed consent form.

### 2.2 Rehabilitation protocol

ACLR patients began their rehabilitation procedure after surgery. Therapists followed a standardized protocol, but blinding was not feasible due to the nature of postoperative rehabilitation. Variability in technique was minimized through regular team training. The specific rehabilitation phases are as follows: Early Phase (0–1 week): The primary focus is on swelling control, pain management, and neuromuscular activation. Ice therapy should be applied every 2 h for 15 min within the first 48 h, followed by 3–5 sessions daily. Ankle pumping exercises (500–1,000 repetitions/day, 5-s holds for dorsiflexion and plantarflexion) are critical for venous return and thrombosis prevention. Passive range of motion (ROM) training begins on postoperative day 1, with heel-supported knee extension (3–5 sessions/day) and bedside leg-dangling flexion (60°–90°, 5 sets of 10 repetitions/day). Quadriceps isometric contractions (10-s holds, 500 repetitions/day) and straight-leg raises (15° elevation, 3 sets of 20 repetitions/day) are initiated to prevent muscle atrophy. A brace locked at 0° is mandatory during ambulation to protect the graft; Initial Phase (2–4 weeks): This phase emphasizes partial weight-bearing transition and balance re-education. Wall-sliding exercises (90°–100° flexion, 6–8 sets of 10 repetitions/day) and patellar mobilization (5 min/session, 5 sessions/day) optimize joint mobility. Strength training includes wall sits (30° knee flexion, 30-s holds, 3 sets of 20 repetitions/day) and lateral step-ups with resistance bands (10 steps/set, 3 sets/day). Weight-bearing progresses from 25% body weight at week 2 to full weight-bearing by week 4. Balance training involves split-leg standing (30 cm apart, 1-min holds, 5 sets/day) and soft-tilt board exercises (15 min cumulative/day); Middle Phase (5 weeks to 3 months): Full ROM restoration (≥120° flexion) and dynamic stability are prioritized. Slide-board-assisted flexion (5° incremental increases/day) and lunge stretches (4–6 breaths/hold, 6 sets/day) enhance terminal ROM. Eccentric hamstring training (Nordic curls: 0°–60° controlled lowering, 3 sets of 15 repetitions/day) reduces anterior tibial shear forces. Advanced proprioception drills include BOSU ball squats with ball throws (20 min/day) and 20-cm step-ups (10 repetitions/set, 3 sets/day). Low-resistance cycling (70–90 rpm, 20–30 min/day) and aquatic walking (chest-deep water, 15 min/day) improve cardiovascular endurance s; Late Phase (4–12 months): This phase prepares for sports-specific demands. Stair training (8–25 cm step height, 2 sets of 15 repetitions/day) and weighted squats (kettlebell-loaded, 90° depth, 3 sets of 10 repetitions/day) build functional strength. Light jogging begins at 6 months (10–15 min/session, 2-3 sessions/week), progressing to 30 min. Agility drills (two-cone figure-eight pattern runs, lateral cross-steps) and sport-specific simulations (jump landings, pivoting) are introduced at 20 min/session, 3 times/week. Functional milestones include ≥85% limb symmetry in single-leg hop tests and <4 cm discrepancy in Y-balance anterior reach (Kotsifaki et al., 2023). We conducted regular follow-up visits and collected gait data and clinical scores at postoperative 3 months, 6 months, and 12 months. Therapists adhered to a standardized protocol, but blinding was infeasible due to the nature of postoperative rehabilitation. Variability in technique was minimized through regular team training.

### 2.3 Knee kinematic data collection

We used a 3D motion capture gait system, Opti_Knee (developed by Innomotion, Shanghai), to collect knee kinematic data. This system uses surgical navigation infrared tracking devices (NDI Polaris Spectra, Northern Digital Inc., Canada), markers, high-speed optical cameras, and handheld digital probes, with a data sampling frequency of 60 Hz. The system has been validated for high accuracy and repeatability (Elfring et al., 2010; Wang et al., 2021; Zhang et al., 2016). Marker clusters were securely attached to the distal thigh and proximal calf using elastic bands to prevent motion artifacts. A handheld probe was used to digitize bony landmarks, including the greater trochanter of the femur, medial/lateral femoral condyles, and medial/lateral malleoli (Zhang et al., 2015). By attaching a set of markers on the patient’s thigh and calf, the system can precisely track the movement of the knee joint. The kinematic data calculation is done by dedicated software (Opti_Knee, developed by Innomotion, Shanghai). The markers identify bony landmarks such as the greater trochanter of the femur, the medial and lateral femoral condyles, and the medial and lateral malleoli, allowing precise recording of the three-dimensional spatial relationship between the femur and tibia. The femur is used as the reference frame, and changes in the tibia’s coordinate system relative to the femoral coordinate system are calculated, with the femur-tibia trajectory collected. Ultimately, we obtained 6DOF knee kinematic data, including flexion-extension (degree), adduction/abduction (degree), femoral internal/external rotation (degree), anterior-posterior translation (mm), proximal/distal translation (mm), and medial/lateral translation (mm) of the knee joint.

Before data collection, all participants underwent an adaptive gait test on a treadmill. The patients were instructed to walk at a comfortable speed, simulating ground walking. Finally, we recorded 15 s of gait data. Averaging 15-s gait data minimizes noise while preserving cycle trends, as validated in prior gait studies (Wang et al., 2021). The total test time for each participant was controlled within 10 min. The gait cycle began when one heel touched the ground and ended when the same heel touched the ground again. In this study, we divided and averaged the gait data for each participant within 15 s to form a complete gait cycle (0%–100% of the gait cycle phase). The gait cycle was divided into the stance phase (0%–62%) and swing phase (62%–100%) (Di Gregorio and Vocenas, 2021).

### 2.4 Clinical scores assessment

We assessed patients using the International Knee Documentation Committee (IKDC) Subjective Knee Evaluation Form and the Knee injury and Osteoarthritis Outcome Score (KOOS) system (Irrgang et al., 2001; Monticone et al., 2012). The assessments were conducted in a quiet clinic environment by researchers who were trained and familiar with the scoring systems. During the assessment, the purpose of the scoring was explained to the patients, and consent was obtained. The researchers then asked the relevant questions and summarized the responses to calculate the total IKDC and KOOS scores.

### 2.5 Statistical analysis

The normality of demographic data (age, height, weight, and body mass index) and clinical scores between groups was assessed using the One-Way ANOVA test with Dunnett’s correction for post-hoc comparisons, setting the control group as the reference. Normality (Shapiro-Wilk test) and homogeneity of variance (Levene’s test) were confirmed (p > 0.05). Non-parametric tests were applied to non-normal data. The gender difference was assessed with a Chi-Squared test. Statistical analysis was conducted in SPSS version 24.0 (IBM Corp., Armonk, NY, United States), with the significance level set at 0.05. Analysts were blinded to group allocation during data processing to reduce measurement bias.

The range of motion of knee kinematics across groups was similarly compared using the One-Way ANOVA test with Dunnett’s correction. Kinematic curve data were analyzed using the One-Way ANOVA test implemented in the one-dimensional statistical parametric mapping (SPM1D) software package (http://spm1d.org). This method applies random field theory and one-dimensional Gaussian smoothing to perform statistical inference on one-dimensional data. SPM1D analysis used cluster-level correction (α = 0.0166) to control for multiple comparisons. For these analyses, the significance level (α) was adjusted to 0.0166 (accounting for three comparisons) based on Dunnett’s correction, with the control group set as the reference.

## 3 Results

### 3.1 Demographic data and clinical scores assessment

At 3 months after ACLR, the group consisted of 24 males and 25 females. At 6 months, there were 16 males and 17 females, and at 12 months, 18 males and 17 females. The control group included 29 males and 29 females. All the demographic characteristics between groups were not significantly different (Table 1).

**TABLE 1:** Demographic Data of ACLR groups and control group.

Variables	3 months	6 months	12 months	Control group	P Value
Male: Female	24:25	16:17	18:17	29:29	0.995
Age (years)	27.7 ± 6.4	26.5 ± 5.8	27.3 ± 6.6	25.7 ± 2.8	0.245
Height (cm)	169.2 ± 7.4	169.8 ± 7.1	169.2 ± 5.9	167.1 ± 8.0	0.283
Weight (kg)	58.5 ± 10.2	59.5 ± 12.5	59.3 ± 11.4	58.2 ± 9.0	0.926
BMI	20.3 ± 2.6	20.6 ± 3.9	20.6 ± 3.1	20.7 ± 2.0	0.916

The knee joint clinical function scores indicated significant improvements in patient function at all postoperative follow-up time points (p < 0.05). The IKDC score significantly increased from 48.8 ± 5.8 at 3 months post-surgery to 66.0 ± 4.7 at 6 months and further improved to 88.2 ± 5.9 at 12 months. The KOOS scores also showed a continuous improvement trend, such as the KOOS Pain score, which increased from 49.0 ± 6.0 at 3 months to 67.6 ± 5.6 at 6 months, and reached 92.0 ± 4.5 at 12 months. These results suggest that the subjective knee joint function and quality of life of ACLR patients significantly improved over time (Table 2).

**TABLE 2:** Knee clinical function scores.

Clinical scores	3 Months	6 Months	12 Months	P Value
IKDC	48.8 ± 5.8	66.0 ± 4.7^a^	88.2 ± 5.9^b^	<0.001
KOOS pain	49.0 ± 6.0	67.6 ± 5.6^a^	92.0 ± 4.5^a^	<0.001
KOOS symptom	57.6 ± 4.7	67.4 ± 4.6^a^	86.7 ± 4.3^a^	<0.001
KOOS ADL	60.5 ± 6.0	76.9 ± 5.7^a^	90.5 ± 3.9^a^	<0.001
KOOS sport	30.4 ± 6.1	50.3 ± 6.5^a^	85.0 ± 5.9^a^	<0.001
KOOS QOL	40.6 ± 6.6	55.3 ± 9.1^a^	87.3 ± 4.9^a^	<0.001

^a^ significant different compared to 3 months.

^b^ significant different compared to 3 months.

### 3.2 Knee kinematic analysis

**FIGURE 1:**
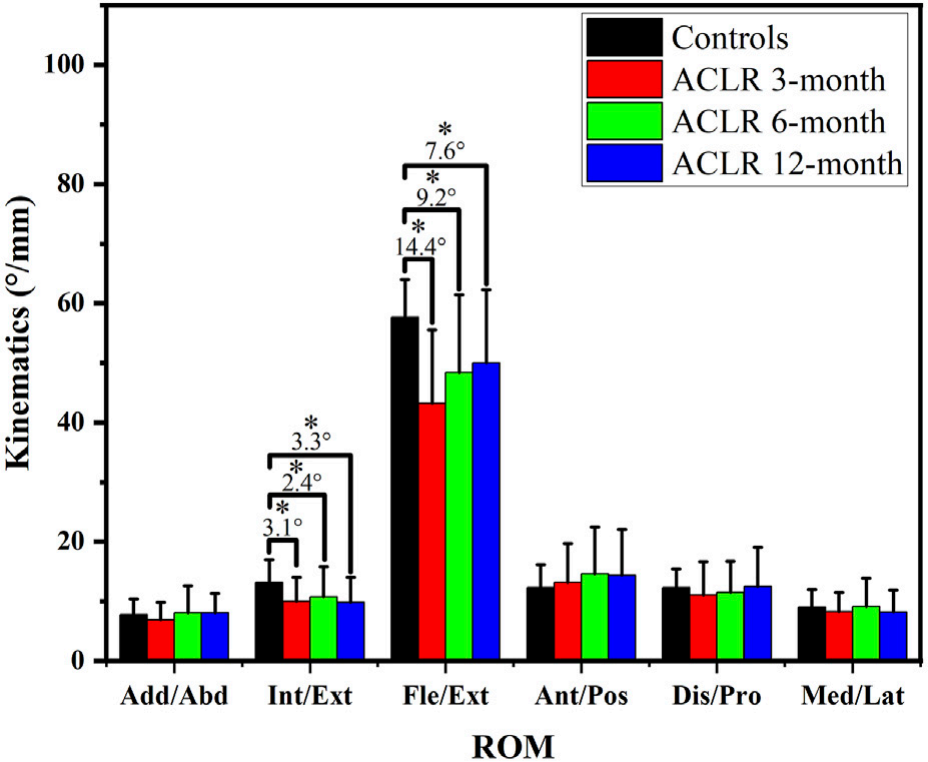
Range of motion of knee kinematics. * significant differences (p < 0.05) between groups.

Throughout the follow-up time points, there were varying degrees of significant differences in the kinematic characteristics of the ACLR knee joint compared to the control group. The range of motion (ROM) of knee kinematics (Figure 1) showed significant differences in internal/external rotation and Flexion/Extension between the control and ACLR groups across the gait cycle. The ROM of internal/external rotation and flexion/extension angles of ACLR groups at different time points were smaller than the control groups (p < 0.05). However, as rehabilitation progressed, at the 12-month follow-up time point, the difference in ACLR kinematics compared to the control group was minimal. Flexion ROM at 12 months differed significantly from 3 months (p = 0.003) but not from 6 months (p = 0.12), indicating a plateau in recovery.

### 3.3 Knee kinematics of the sagittal plane

**FIGURE 2:**
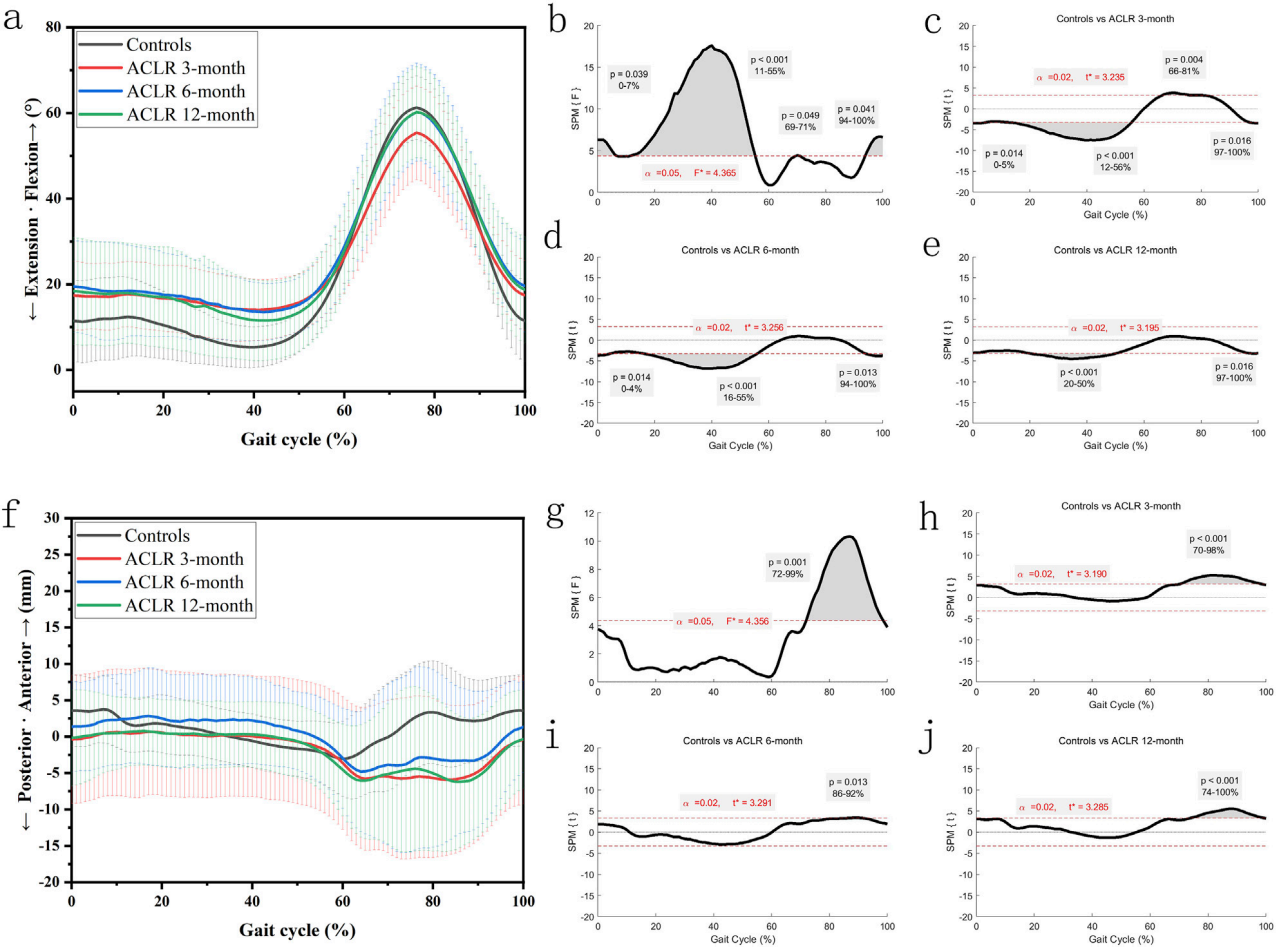
Knee kinematics of the sagittal plane in a gait cycle. Chart **(a)**, flexion/extension angles in a gait cycle; chart **(b)**, F value of flexion/extension using SPM1D method (One Way ANOVA section) in a gait cycle; chart **(c)**, posthoc statistical comparison of flexion/extension between the control group and ACLR patients at 3 months; chart **(d)**, posthoc statistical comparison of flexion/extension between the control group and ACLR patients at 6 months; chart **(e)**, posthoc statistical comparison of flexion/extension between the control group and ACLR patients at 12 months; Chart **(f)**, anterior/posterior tibial translation in a gait cycle; chart **(g)**, F value of anterior/posterior tibial translation using SPM1D method (One Way ANOVA section) in a gait cycle; chart **(h)**, posthoc statistical comparison of anterior/posterior tibial translation between the control group and ACLR patients at 3 months; chart **(i)**, posthoc statistical comparison of anterior/posterior tibial translation between the control group and ACLR patients at 6 months; chart **(j)**, posthoc statistical comparison of anterior/posterior tibial translation between the control group and ACLR patients at 12 months; the posthoc comparison of SPM1D method was based on Dunnett tests.

The knee extension-flexion angles (Figures 2a–e) showed significant differences between the control and ACLR groups across the gait cycle. SPM analysis (Figures 2b–e) revealed significant differences at 3 months (p < 0.001 from 12% to 56% and p = 0.004 from 66% to 81%, Figure 2c), at 6 months (p < 0.001 from 16% to 55% and p = 0.013 from 94% to 100%, Figure 2d), and at 12 months (p < 0.001 from 20% to 50% and p = 0.016 from 97% to 100%, Figure 2e). These findings suggest partial recovery of knee extension-flexion kinematics by 12 months post-ACLR.

The anterior tibial translation (Figures 2f–j) showed significant differences between the control and ACLR groups across the gait cycle. SPM analysis (Figures 2g–j) revealed significant differences at 3 months (p < 0.001 from 70% to 98%, Figure 2h), at 6 months (p = 0.013 from 86% to 92%, Figure 2i), and at 12 months (p < 0.001 from 74% to 100%, Figure 2j). These findings suggest no significant extra-changes of anterior tibial translation by 12 months post-ACLR. Increased anterior translation at 12 months may reflect graft laxity or delayed neuromuscular fatigue, necessitating biomechanical imaging for validation.

### 3.4 Knee kinematics of the transverse plane

**FIGURE 3:**
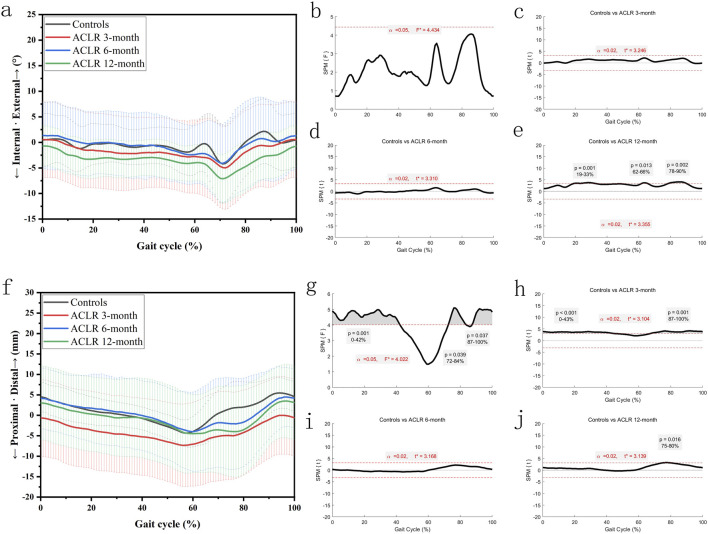
Knee kinematics of the transverse plane in a gait cycle. Chart **(a)**, internal/external rotation angles in a gait cycle; chart **(b)**, F value of internal/external rotation using SPM1D method (One Way ANOVA section) in a gait cycle; chart **(c)**, posthoc statistical comparison of internal/external rotation between the control group and ACLR patients at 3 months; chart **(d)**, posthoc statistical comparison of internal/external rotation between the control group and ACLR patients at 6 months; chart **(e)**, posthoc statistical comparison of internal/external rotation between the control group and ACLR patients at 12 months; Chart **(f)**, distal/proximal tibial translation in a gait cycle; chart **(g)**, F value of distal/proximal tibial translation using SPM1D method (One Way ANOVA section) in a gait cycle; chart **(h)**, posthoc statistical comparison of distal/proximal tibial translation between the control group and ACLR patients at 3 months; chart **(i)**, posthoc statistical comparison of distal/proximal tibial translation between the control group and ACLR patients at 6 months; chart **(j)**, posthoc statistical comparison of distal/proximal tibial translation between the control group and ACLR patients at 12 months; the posthoc comparison of SPM1D method was based on Dunnett tests.

The knee internal-external rotation angles (Figures 3a–e) showed significant differences between the control and ACLR groups across the gait cycle. SPM analysis (Figures 3b–e) revealed differences at all recovery stages, with p = 0.001 for the entire gait cycle at 3 months (Figure 3c). At 6 months, significant differences (Figure 3d) were observed from 19% to 33% (p = 0.001) and 62%–66% (p = 0.013). At 12 months, differences (Figure 3e) were significant from 19% to 33% (p = 0.001), 62%–66% (p = 0.013), and 78%–90% (p = 0.002). These findings suggest partial normalization of knee rotation by 12 months post-ACLR.

The distal tibial translation (Figures 3f–j) showed significant differences between the control and ACLR groups across the gait cycle. SPM analysis (Figures 3g–j) revealed significant differences at 3 months (p < 0.001 from 0% to 43%% and p = 0.001 from 87% to 100%, Figure 3h). There was little significant difference at 6 months and 12 months (p = 0.016 from 75% to 80%, Figure 3j). These findings suggest great recovery of distal tibial translation kinematics by 12 months post-ACLR.

### 3.5 Knee kinematics of the coronal plane

**FIGURE 4:**
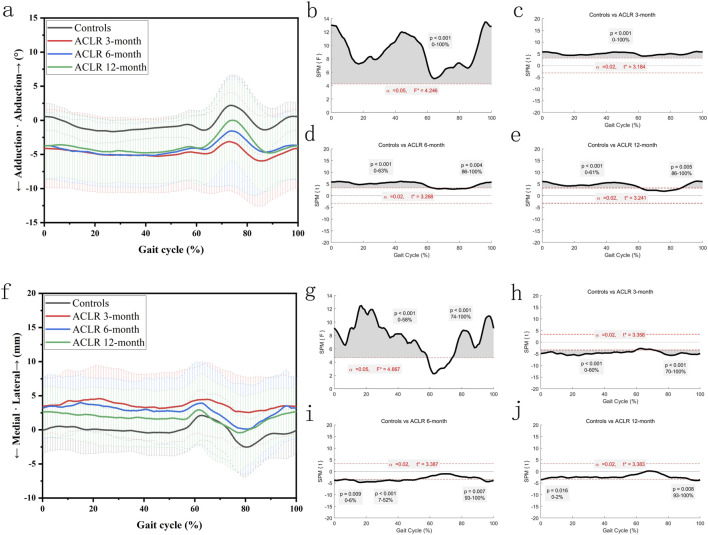
Knee kinematics of the coronal plane in a gait cycle. Chart **(a)**, adduction/abduction angles in a gait cycle; chart **(b)**, F value of adduction/abduction using SPM1D method (One Way ANOVA section) in a gait cycle; chart **(c)**, posthoc statistical comparison of adduction/abduction between the control group and ACLR patients at 3 months; chart **(d)**, posthoc statistical comparison of adduction/abduction between the control group and ACLR patients at 6 months; chart **(e)**, posthoc statistical comparison of adduction/abduction between the control group and ACLR patients at 12 months; Chart **(f)**, medial/lateral tibial translation in a gait cycle; chart **(g)**, F value of medial/lateral tibial translation using SPM1D method (One Way ANOVA section) in a gait cycle; chart **(h)**, posthoc statistical comparison of medial/lateral tibial translation between the control group and ACLR patients at 3 months; chart **(i)**, posthoc statistical comparison of medial/lateral tibial translation between the control group and ACLR patients at 6 months; chart **(j)**, posthoc statistical comparison of medial/lateral tibial translation between the control group and ACLR patients at 12 months; the posthoc comparison of SPM1D method was based on Dunnett tests.

Figure 4 showed significant differences in knee adduction-abduction angles and distal tibial translation between the control and ACLR groups across the gait cycle. SPM analysis (Figures 4a–e) revealed significant deviations of knee adduction-abduction angles at all recovery stages (3, 6, and 12 months), with p-values <0.001 for the 0%–100% gait cycle at 3 months (Figure 4c). At 6 months, differences (Figure 4d) were significant from 0% to 63% (p < 0.001) and 86%–100% (p = 0.004), while at 12 months, differences (Figure 4e) were significant from 0% to 61% (p < 0.001) and 86%–100% (p = 0.005).

SPM analysis (Figures 4f –j) revealed significant differences in medial tibial translation at 3 months (p < 0.001 from 0% to 60% and p < 0.001 from 70% to 100%, Figure 4h), at 6 months (p = 0.009 from 0% to 6%, p < 0.001 from 7% to 52%, and p = 0.007 from 93% to 100%, Figure 4i), and at 12 months (p = 0.016 from 0% to 2% and p = 0.008 from 93% to 100%, Figure 4j). These findings suggest recovery of distal tibial translation kinematics by 12 months post-ACLR. These differences diminished as recovery progressed, with partial normalization by 12 months post-ACLR.

## 4 Discussion

This study demonstrates that the IKDC and KOOS scores of ACLR patients significantly improved between 3 and 12 months post-surgery, which is consistent with the findings of Everhart et al. (2022). Their 20-year follow-up study on ACLR patients found that the IKDC scores were generally close to or at normal levels, indicating that ACLR surgery has a significant effect on improving patients' quality of life and subjective evaluations (Everhart et al., 2022). However, despite the significant improvement, other studies have indicated that objective knee function recovery, particularly the restoration of knee biomechanics, still presents challenges within the first year post-surgery (Knurr et al., 2021; Zhou et al., 2022). This suggests that although clinical scores improve, the recovery of knee biomechanics in patients may still be far from ideal, reflecting the complexity and variability of postoperative rehabilitation.

ACLR patients demonstrated significantly smaller adduction angles compared to healthy controls at 6 months postoperatively, potentially indicating transient biomechanical overcorrection during early rehabilitation. However, these differences resolved by 12 months, suggesting gradual normalization of gait patterns. Previous studies have shown that changes in the peak pressure within the medial knee compartment are closely related to increases in the adduction angle after ACLR (Wellsandt et al., 2016). This result suggests that the abnormal adduction angle in ACLR patients may be a potential risk factor for early knee joint degeneration or the development of osteoarthritis. Therefore, adjustments and monitoring of the adduction angle in the early postoperative stage, especially within 6 months, are crucial. This discrepancy may be attributed to progressive neuromuscular compensatory mechanisms—early deviations (e.g., internal rotation asymmetry) could be gradually mitigated through reconstructed quadriceps-hamstrings co-activation patterns, restored proprioception, and adaptive gait strategies.

In addition, ACLR patients exhibited notable changes in the internal/external rotation angles between 6 and 12 months, particularly an increase in the internal rotation angle. Literature has already pointed out that abnormal internal rotation angles after ACLR may be closely related to the onset of knee joint degeneration, such as knee osteoarthritis (Rodriguez-Merchan and Encinas-Ullan, 2022; Scanlan et al., 2010). This abnormal internal rotation angle may cause uneven loading on the knee joint during the gait cycle, thus increasing the risk of cartilage degeneration. Therefore, early identification and correction of these internal rotation abnormalities are of great significance in preventing the development of postoperative knee osteoarthritis.

During the stance phase of the gait cycle, ACLR patients showed significant impairment in knee extension, which was particularly prominent at 6 months post-surgery. Although some improvement was observed at 12 months, full recovery was not achieved. Restoration of knee extensor strength is one of the key goals in ACLR rehabilitation. Studies have shown that deficits in knee extensor strength are closely related to decreased knee function, increased risk of osteoarthritis, and recurrence of sports injuries (Buckthorpe et al., 2019; Hart et al., 2016; Lisee et al., 2022). Therefore, early strengthening exercises for the knee extensors can not only improve function but may also play a key role in preventing the progression of knee osteoarthritis.

On the other hand, ACLR patients exhibited significant increased posterior tibial displacement during the swing phase of the gait cycle. Although this abnormality improved at 6 months, it reappeared at 12 months post-surgery. The transient increase in anterior translation at 12 months could represent physiological graft maturation rather than pathological laxity. The tibial displacement difference (1.2 mm) is close to the system error (±1 mm), so the results should be interpreted with caution. Additionally, the abnormal medial-lateral displacement of the knee persisted until 12 months post-surgery, with gradual improvement over time, but still presenting challenges for full recovery. Recurrent displacement at 12 months may reflect graft creep or delayed neuromuscular control (Sherman et al., 2021). Abnormal knee kinematics may alter the distribution of cartilage load, increasing the risk of cartilage wear and knee joint degeneration.

It is worth noting that the recovery of distal-proximal displacement in ACLR patients was nearly complete within 3 months post-surgery, and the recovery of quadriceps strength was closely related to improvements in knee joint space (Pietrosimone et al., 2019). This finding suggests that postoperative quadriceps strengthening exercises can effectively improve the biomechanical performance of the knee joint, especially in restoring joint space, providing important intervention support for postoperative rehabilitation.

The following is our correction plan for improving the abnormal biomechanical parameters after ACLR. Real-time biofeedback training (threshold: adduction angle ≤3°) within 6 months postoperatively. Eccentric quadriceps strengthening (initiated at 4 weeks postoperatively): Single-leg eccentric squats performed twice weekly with progressive loading. Functional electrical stimulation for patients with Vastus Medialis Oblique activation deficits. This strategy aims to correct abnormal mechanical loading and reduce osteoarthritis risk.

In conclusion, this study demonstrates that the recovery of knee joint function in ACLR patients shows stage-dependent differences, with varying recovery patterns across different biomechanical parameters. While this study identified persistent kinematic abnormalities during the first 12 months following ACL reconstruction, a 20-year longitudinal follow-up study demonstrated normalized IKDC scores in long-term outcomes (Everhart et al., 2022), implying potential temporal resolution of initial movement impairments. This apparent contradiction between short-term observations and long-term functional recovery highlights the need for extended longitudinal monitoring to elucidate the trajectory of kinematic recovery and validate the neuromuscular compensation hypothesis.

To advance these insights, future investigations should: (1) Implement longitudinal designs with extended 5-year follow-ups to map OA progression against residual kinematic deficits; (2) Employ within-subject comparisons (operative vs. contralateral limbs) to detect individualized recovery patterns; (3) Integrate biplanar fluoroscopy for precise graft biomechanics analysis during functional movements; (4) Validate real-time biofeedback protocols targeting persistent rotational abnormalities. Such methodological enhancements will improve causal inference while informing personalized rehabilitation frameworks.

## 5 Conclusion

This study reveals the dynamic changes and recovery patterns of knee joint six degrees of freedom kinematics within 1 year after ACL reconstruction. Clinical function scores of patients significantly improved between 3 and 12 months post-surgery, but gait kinematics exhibited abnormalities in several degrees of freedom, with significant differences in the recovery process. Specifically, the adduction-abduction angle and anterior-posterior displacement showed significant improvement within 6 months post-surgery, stabilizing afterward but not fully recovering. Abnormalities in internal-external rotation and flexion-extension angles worsened or showed no significant improvement between 6 and 12 months post-surgery, suggesting that later-stage rehabilitation should focus on optimizing rotational control and flexion-extension function. Additionally, distal-proximal displacement was nearly fully recovered within 3 months post-surgery, while abnormal lateral displacement persisted until 12 months but gradually improved. The findings provide scientific evidence for the optimization of individualized rehabilitation strategies and emphasize the importance of correcting key kinematic abnormalities for functional recovery in later-stage rehabilitation.

## Data Availability

The data analyzed in this study is subject to the following licenses/restrictions: Because of patient privacy restrictions, the data in this report will not be made public. Requests for the data presented in this study are available from the corresponding authors. Requests to access these datasets should be directed to TZ, gzlupus@126.com.
