# Supplementary material for: The evolution of three-dimensional knee kinematics after ACL reconstruction within one year
Source: Front Bioeng Biotechnol. 2025 Apr 23;13:1572160. doi: 10.3389/fbioe.2025.1572160 (PMC12055532; doi:10.3389/fbioe.2025.1572160)
Supplement: Supplementary file 1 [file Table1.DOCX]

Supplementary Material

**Table 1. Demographic Data of ACLR groups and control group.**

| **Variables** | **3 months** | **6 months** | **12 months** | **Control Group** | **P value** |
| --- | --- | --- | --- | --- | --- |
| **Male: Female** | 24:25 | 16:17 | 18:17 | 29:29 | 0.995 |
| **Age (years)** | 27.7±6.4 | 26.5±5.8 | 27.3±6.6 | 25.7±2.8 | 0.245 |
| **Height (cm)** | 169.2±7.4 | 169.8±7.1 | 169.2±5.9 | 167.1±8.0 | 0.283 |
| **Weight (kg)** | 58.5±10.2 | 59.5±12.5 | 59.3±11.4 | 58.2±9.0 | 0.926 |
| **BMI** | 20.3±2.6 | 20.6±3.9 | 20.6±3.1 | 20.7±2.0 | 0.916 |

**Table 2 Knee Clinical Function Scores.**

| Clinical Scores | 3 Months | 6 Months | 12 Months | P value |
| --- | --- | --- | --- | --- |
| IKDC | 48.8±5.8 | 66.0±4.7^*^ | 88.2±5.9^#^ | <0.001 |
| KOOS pain | 49.0±6.0 | 67.6±5.6^*^ | 92.0±4.5^*^ | <0.001 |
| KOOS symptom | 57.6±4.7 | 67.4±4.6^*^ | 86.7±4.3^*^ | <0.001 |
| KOOS ADL | 60.5±6.0 | 76.9±5.7^*^ | 90.5±3.9^*^ | <0.001 |
| KOOS sport | 30.4±6.1 | 50.3±6.5^*^ | 85.0±5.9^*^ | <0.001 |
| KOOS QOL | 40.6±6.6 | 55.3±9.1^*^ | 87.3±4.9^*^ | <0.001 |

*significant different compared to 3 months

#significant different compared to 3 months


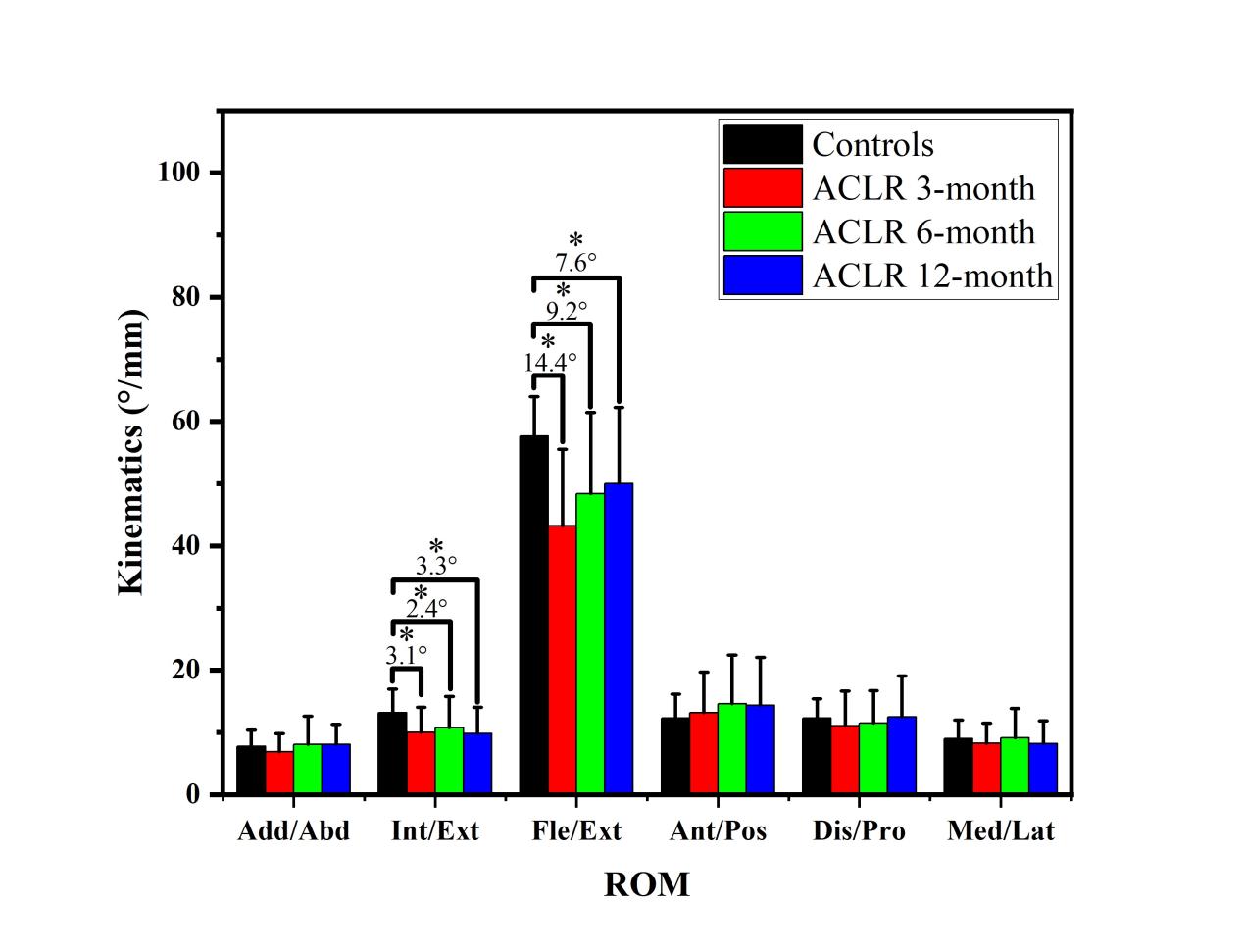


**Figure 1. Range of motion of knee kinematics.** * significant differences (p<0.05) between groups.


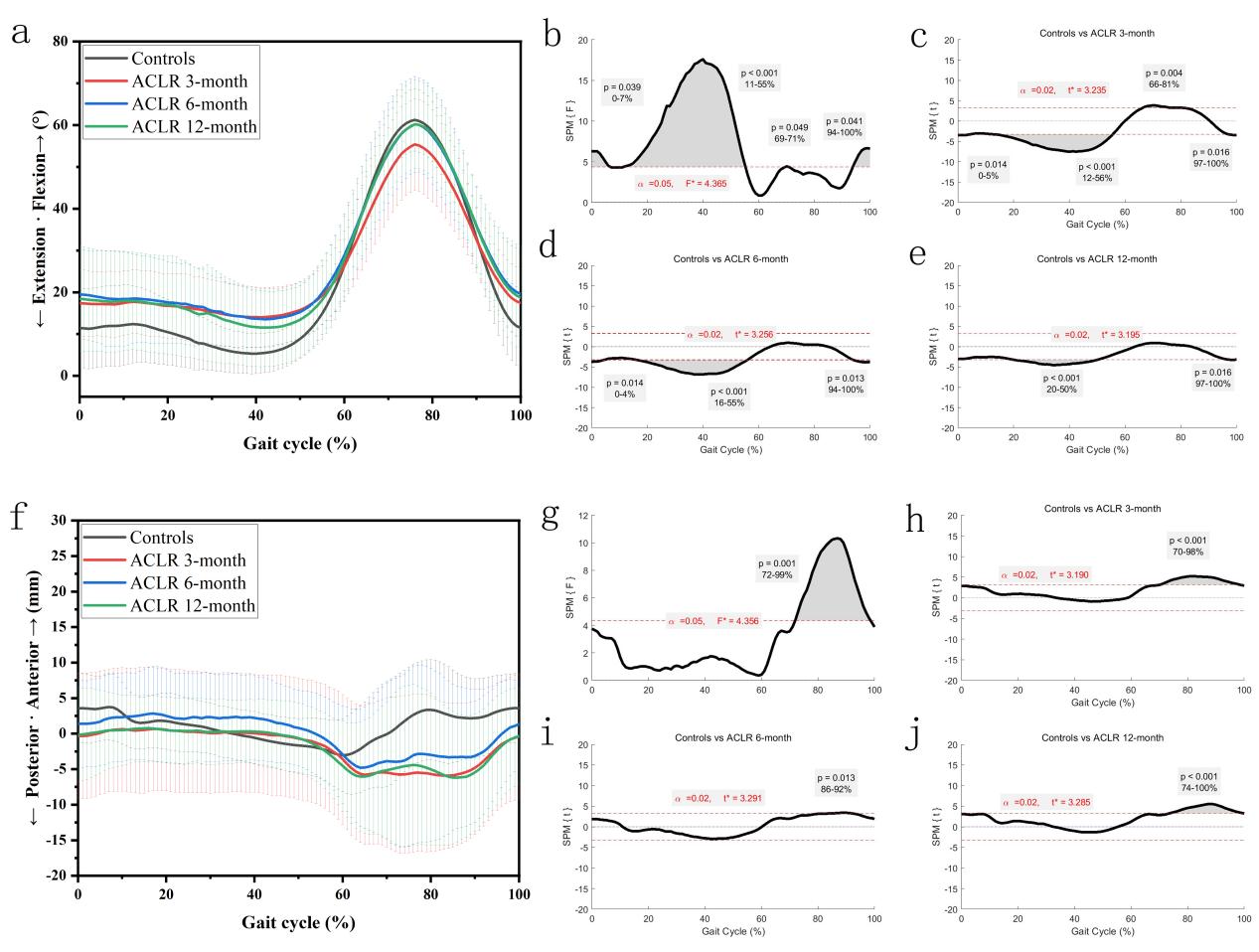


**Figure 2. Knee kinematics of the sagittal plane in a gait cycle.** Chart a, flexion/extension angles in a gait cycle; chart b, F value of flexion/extension using SPM1D method (One Way ANOVA section) in a gait cycle; chart c, posthoc statistical comparison of flexion/extension between the control group and ACLR patients at 3 months; chart d, posthoc statistical comparison of flexion/extension between the control group and ACLR patients at 6 months; chart e, posthoc statistical comparison of flexion/extension between the control group and ACLR patients at 12 months; Chart f, anterior/posterior tibial translation in a gait cycle; chart g, F value of anterior/posterior tibial translation using SPM1D method (One Way ANOVA section) in a gait cycle; chart h, posthoc statistical comparison of anterior/posterior tibial translation between the control group and ACLR patients at 3 months; chart i, posthoc statistical comparison of anterior/posterior tibial translation between the control group and ACLR patients at 6 months; chart j, posthoc statistical comparison of anterior/posterior tibial translation between the control group and ACLR patients at 12 months; the posthoc comparison of SPM1D method was based on Dunnett tests.


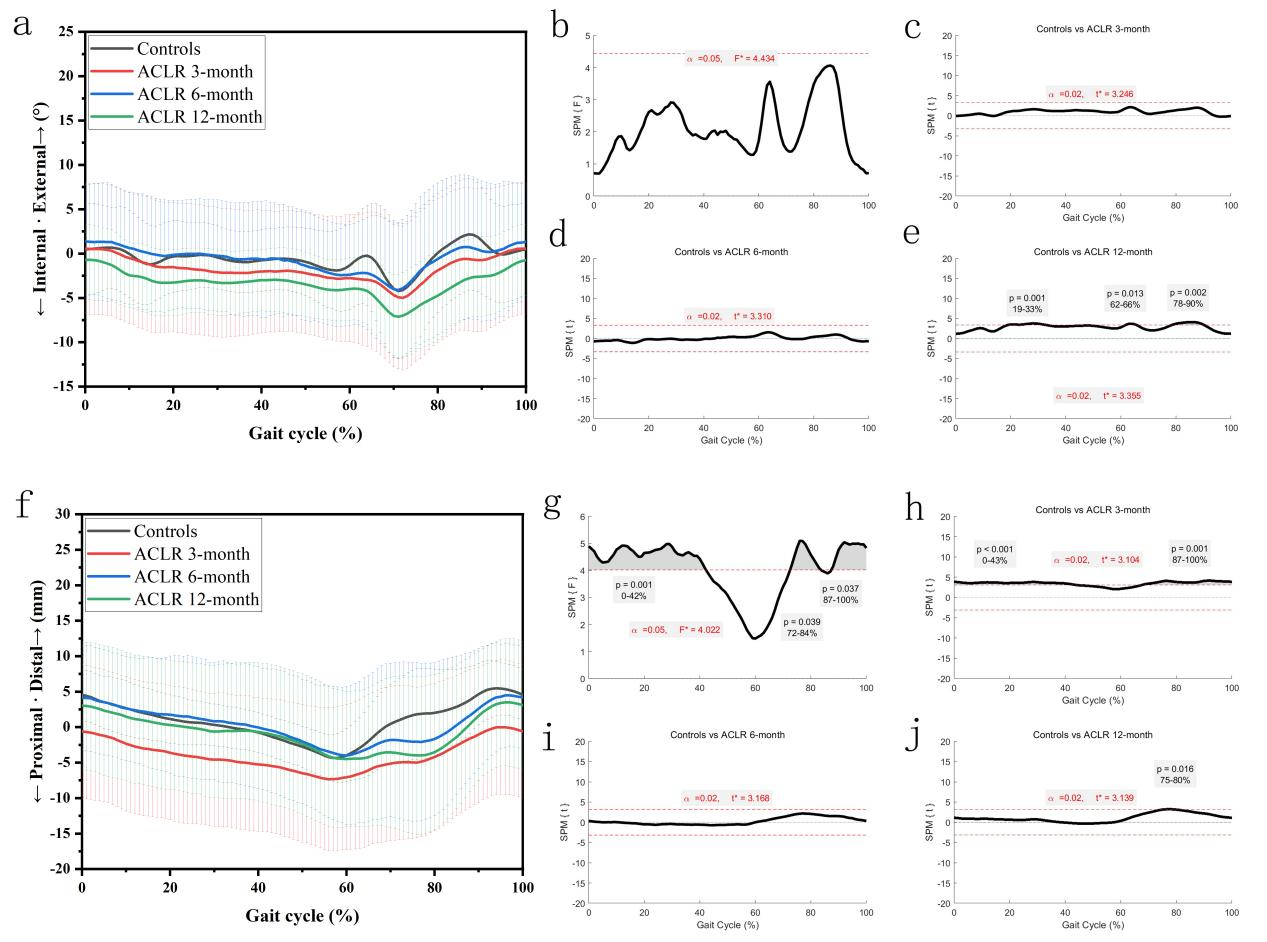


**Figure 3. Knee kinematics of the transverse plane in a gait cycle.** Chart a, internal/external rotation angles in a gait cycle; chart b, F value of internal/external rotation using SPM1D method (One Way ANOVA section) in a gait cycle; chart c, posthoc statistical comparison of internal/external rotation between the control group and ACLR patients at 3 months; chart d, posthoc statistical comparison of internal/external rotation between the control group and ACLR patients at 6 months; chart e, posthoc statistical comparison of internal/external rotation between the control group and ACLR patients at 12 months; Chart f, distal/proximal tibial translation in a gait cycle; chart g, F value of distal/proximal tibial translation using SPM1D method (One Way ANOVA section) in a gait cycle; chart h, posthoc statistical comparison of distal/proximal tibial translation between the control group and ACLR patients at 3 months; chart i, posthoc statistical comparison of distal/proximal tibial translation between the control group and ACLR patients at 6 months; chart j, posthoc statistical comparison of distal/proximal tibial translation between the control group and ACLR patients at 12 months; the posthoc comparison of SPM1D method was based on Dunnett tests.

**
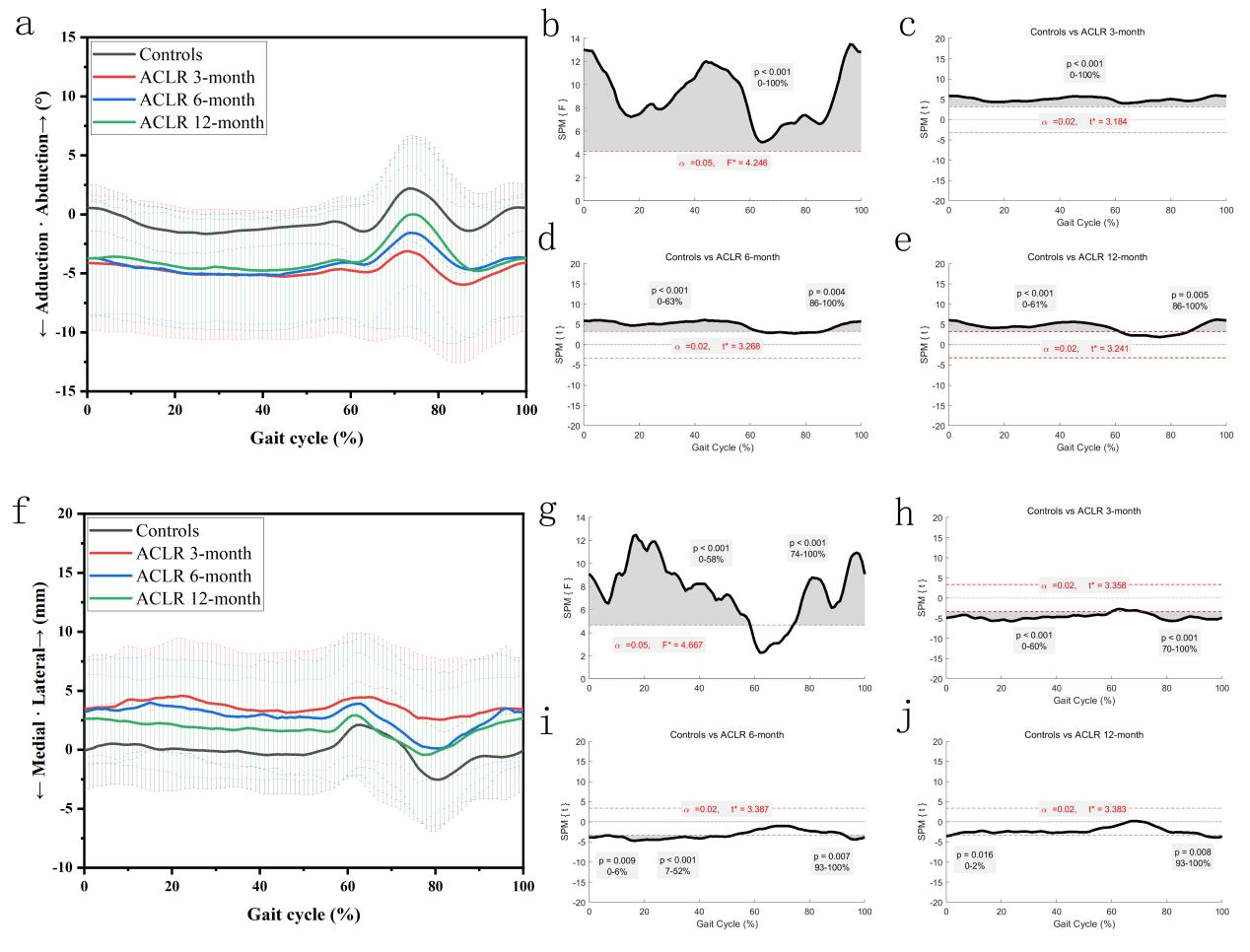
**

**Figure 4. Knee kinematics of the coronal plane in a gait cycle.** Chart a, adduction/abduction angles in a gait cycle; chart b, F value of adduction/abduction using SPM1D method (One Way ANOVA section) in a gait cycle; chart c, posthoc statistical comparison of adduction/abduction between the control group and ACLR patients at 3 months; chart d, posthoc statistical comparison of adduction/abduction between the control group and ACLR patients at 6 months; chart e, posthoc statistical comparison of adduction/abduction between the control group and ACLR patients at 12 months; Chart f, medial/lateral tibial translation in a gait cycle; chart g, F value of medial/lateral tibial translation using SPM1D method (One Way ANOVA section) in a gait cycle; chart h, posthoc statistical comparison of medial/lateral tibial translation between the control group and ACLR patients at 3 months; chart i, posthoc statistical comparison of medial/lateral tibial translation between the control group and ACLR patients at 6 months; chart j, posthoc statistical comparison of medial/lateral tibial translation between the control group and ACLR patients at 12 months; the posthoc comparison of SPM1D method was based on Dunnett tests.
